# Afamin: an early predictor of preeclampsia

**DOI:** 10.1007/s00404-018-4897-z

**Published:** 2018-09-15

**Authors:** Angela Köninger, Antje Enekwe, Pawel Mach, Dimitrios Andrikos, Boerge Schmidt, Mirjam Frank, Cahit Birdir, Rainer Kimmig, Alexandra Gellhaus, Hans Dieplinger

**Affiliations:** 1 Department of Gynaecology and Obstetrics, University of Duisburg-Essen, Hufelandstrasse 55, 45122 Essen, Germany; 2 Institute for Medical Informatics, Biometry and Epidemiology (IMIBE), University of Duisburg-Essen, Hufelandstraße 55, 45122 Essen, Germany; 3 Department of Gynaecology and Obstetrics, University Hospital Carl Gustav Carus, Fletscherstraße 74, 01307 Dresden, Germany; 4 Division of Genetic Epidemiology, Department of Medical Genetics, Molecular and Clinical Pharmacology, Medical University of Innsbruck, Schöpfstraße 41, 6020 Innsbruck, Austria

**Keywords:** Afamin, Preeclampsia, Early and late onset, Prediction

## Abstract

**Purpose:**

Oxidative stress is involved in the pathogenesis of hypertensive disorders such as preeclampsia (PE) and associated with the human vitamin E-binding protein afamin. The aim of this study was, therefore, to analyse afamin in the first trimester of patients developing PE later in pregnancy and in control subjects without pregnancy complications.

**Methods:**

In this retrospective study, 137 serum samples from the first trimester of pregnancy were analysed in a case–control study design. 39 patients developed PE (10 patients with early-onset and 29 patients with late onset disease) and 98 women had an uncomplicated pregnancy. Mann–Whitney *U* test, *t* test, logistic regression and ROC analyses were performed for statistical evaluation.

**Results:**

Pregnant women developing PE presented with higher afamin concentrations in the first trimester [median 101.81 mg/L; interquartile range (IQR) 88.94–113.26] compared to subjects with uncomplicated pregnancy (median 86.40; IQR 75.26–96.92; *p* < 0.001). After adjusting for confounders, the odds ratio per afamin standard deviation was 1.60 (95% CI: 1.04–2.58; *p* = 0.04). An afamin threshold concentration of 87.8 mg/L exhibited the best sensitivity (79.5%) and specificity (57.1%) in predicting PE. Subgroup analysis of early- and late-onset disease resulted in substantially higher afamin concentrations in women with developing late-onset PE compared to controls (*p* < 0.001) with an odds ratio per afamin standard deviation of 1.62 (95% CI: 0.98–2.70; *p* = 0.06).

**Conclusions:**

Serum afamin concentrations are elevated in the first trimester among patients developing PE compared to controls. Substantial differences were observed mainly among patients with late-onset PE.

## Introduction

Preeclampsia (PE) and other hypertensive disorders during pregnancy occur in ~ 2–8% of pregnant women [1]. Currently, no treatment exists to prevent the progression of the disease after its clinical diagnosis. However, the occurrence of placental disorders can be prevented by the administration of Aspirin^®^ before the 16th week of pregnancy in women with clinical risk factors for PE such as pathological Doppler indices, positive family history or with PE in a previous pregnancy [2] as well as in primigravidae screened to be at high risk [3]. Elevated risk can be determined in the first trimester by screening for the biochemical markers, pregnancy-associated plasma protein A (PAPP-A) and placental growth factor (PlGF), as well as for biophysical markers such as maternal mean arterial blood pressure and pulsatility indices of the uterine arteries [4, 5].

PE is characterised as an early-onset form, which occurs before the 34th week of pregnancy, and a late-onset form, which occurs after 34 weeks of pregnancy. The prediction [4] as well as prevention [2, 3] of early-onset PE is much more successful compared to the late-onset form suggesting different pathophysiological mechanisms [6].

While early-onset PE is thought to result from disturbed trophoblast invasion, the late-onset form generally seems to result from adverse maternal conditions [7]. In summary, reliable tools have been established to screen and prevent early-onset PE, whereas the late-onset form still needs further research regarding prediction and prevention.

For this reason, our research focused on maternal conditions which may contribute to the pathophysiology leading to placental dysfunction and PE, mainly in the late-onset form. It is already well known that PE is strongly associated with a maternal state of endothelial dysfunction, chronic inflammation, and oxidative stress [8–11]. Women suffering from placental dysfunction subsequently develop metabolic syndrome and insulin resistance (IR) at a frequency of 20 and 60%, respectively [8].

In the present work, we have investigated the role of afamin in the development of PE. Afamin has been previously described as a vitamin E-binding glycoprotein from human plasma which is also found in extravascular fluids [12]. It is a member of the albumin gene family [13]. Vitamin E is an antioxidant nutrient that prevents the oxidation of lipids [14]. In contrast to human plasma, a substantial association between afamin concentrations and vitamin E concentrations has been detected in extravascular fluids like cerebrospinal and follicular fluids [15]. It has been previously reported that serum afamin concentrations are increased in response to various conditions of oxidative stress: afamin concentrations are elevated in the peritoneal fluid of women with endometriosis [16], and are strongly associated with the development of metabolic syndrome including a strong correlation with body mass index (BMI) [17]. A recently published population-based study including more than 20,000 individuals demonstrated elevated afamin concentrations in individuals with type 2 diabetes mellitus (T2DM) and IR [18]. Serum afamin concentrations are elevated in patients with polycystic ovarian syndrome (PCOS) and IR [19, 20] and in the first trimester of women developing subsequent gestational diabetes mellitus [21, 22]. These observations agree with the results of several studies that have suggested cross-links between oxidative stress, the insulin pathway, chronic inflammation and endothelial dysfunction in the pathogenesis of PE [23–26]. Afamin concentrations are known to rise continuously with increasing gestational age [27]. Since afamin is not synthesised by the placenta [27], its concentrations reflect most likely a maternal condition rather than a placental disease.

The aim of this study was to evaluate a possible association between PE, especially in the late-onset form, and concentrations of afamin as an indicator of oxidative stress by comparing afamin concentrations during the first trimester in the serum of pregnant women subsequently developing PE with matched healthy controls without pregnancy complications.

## Materials and methods

### Study design and subjects

Patients and control subjects were analysed retrospectively in a nested case–control study design (pre-eclamptic patients: *n* = 39; controls: *n* = 98) recruited from a consecutively performed first-trimester screening programme at the Department of Obstetrics and Gynaecology of the University Hospital of Essen for obstetric care between 2002 and 2012. Patients and healthy controls were selected from available stored blood samples.

Blood samples were collected between week 11 + 0 and week 13 + 6 of pregnancy. All patients had singleton pregnancies. Patients with gestational diabetes, pre-existing diabetes, chromosomal anomalies or preterm delivery except because of PE were excluded from the study. PE was defined according to the guidelines of the American College of Obstetricians and Gynecologists for hypertension in pregnancy, i.e. proteinuria of ≥ 300 mg/24 h and elevated blood pressure with diastolic values ≥ 90 mmHg and systolic values ≥ 140 mg (measured at least twice within 4–6 h) after the 20th week of pregnancy [28]. Controls were selected from pregnant subjects who did not develop PE or related outcomes during pregnancy.

From the 39 patients who developed PE during the course of pregnancy, 10 patients had early-onset PE and 29 patients had late-onset PE. Patient characteristics are shown in Table 1.

**Table 1:** Characteristics of patients with preeclampsia (PE) and patients with uncomplicated pregnancies (determined during the first trimester)

Mean (STD) Median (IQR)	Control subjects (*N* = 98)	PE (*N* = 39)	*p* value	Early-onset PE (*N* = 10)	Late-onset PE (*N* = 29)	*p* value
Age (years)	32.79 (4.81) 33.00 (29.00–37.00)	33.15 (4.97) 34.00 (31.00–37.00)	0.69	30.50 (5.58) 31.00 (30.00–33.00)	34.07 (4.48) 35.00 (31.00–37.00)	0.05
Afamin (mg/L)	86.90 (17.06) 86.40 (75.26–96.92)	103.13 (23.58) 101.81 (88.94–113.26)	< 0.001	95.56 (16.45) 93.68 (88.03–102.00)	105.75 (25.31) 105.10 (89.78–116.17)	0.20
PAPP-A (ng/mL)	1.29 (0.72) 1.13 (0.74–1.76)	1.70 (2.05) 1.13 (0.65–1.79)	0.84	0.88 (0.59) 0.80 (0.62–1.11)	1.99 (2.30) 1.28 (0.68–2.33)	0.06
PlGF (pg/mL)	41.97 (18.59) 37.65 (29.53–48.39)	30.75 (18.40) 27.08 (19.07–36.05)	< 0.001	29.86 (22.61) 24.43 (18.76–36.27)	31.05 (17.17) 28.38 (20.15–35.90)	0.71
BMI (kg/m^2^)	23.91 (4.95) 22.90 (20.90–25.00)	30.45 (7.40) 29.00 (23.10–36.78)	< 0.001	29.58 (7.53) 28.85 (23.10–35.00)	30.75 (7.47) 29.00 (23.08–37.53)	0.85
Gestational age at blood sampling (d)	87 (4) 87 (85–90)	88 (5) 88 (85–91)	0.19	89 (6) 87 (85–95)	88 (4) 88 (86–91)	0.53
Gestational age at delivery (d, weeks + d)	274 (8) 39 + 1 (1 + 1) 274 (268–281) 39 + 1 (38 + 2–40 + 1)	249 (28) 35 + 4 (4 + 0) 256 (245–266) 36 + 4 (35 + 0–38 + 0)	< 0.001	214 (34) 30 + 4 (4 + 6) 214 (181–236) 30 + 4 (25 + 6–33 + 5)	261 (11) 37 + 2 (1 + 4) 257 (253–268) 36 + 5 (36 + 1–38 + 2)	< 0.001
Birth weight (g)	3417 (425) 3378 (3150–3640)	2604 (938) 2860 (2286–3258)	< 0.001	1388 (966) 1345 (450–2030)	3024 (430) 2985 (2714–3429)	< 0.001
Birth weight (percentiles) [41]	50.21 (25.27) 51.50 (31.00–69.00)	42.46 (28.31) 42.00 (18.50–61.00)	0.13	24.90 (32.58) 12.50 (1.00–36.00)	48.52 (24.46) 51.00 (26.75–64.25)	0.02

Values are presented as means with standard deviations (STD); medians with interquartile ranges (IQR)

*BMI* body mass index, *PAPP-A* pregnancy-associated plasma protein A, *PlGF* placental growth factor, *d* days

### Sample collection and laboratory parameters

9 ml of blood was taken from every patient with the S-Monovette Blood Collection System (Sarstedt AG and Co., Nürnbrecht, Germany) for subsequent determination of parameters of interest. Blood samples were immediately stored at 4 °C and processed within 4 h to avoid cell lysis. Blood fractionation was carried out by centrifugation at 2500×*g* for 10 min, and 3–4 ml of the supernatant constituting blood serum was removed and stored at − 80 °C. For afamin analysis, the samples were thawed, divided into aliquots and restored at − 80 °C. Frozen aliquots were sent to the Division of Genetic Epidemiology at Medical University of Innsbruck in 2016 for analysing afamin. Afamin concentrations were measured with a double-antibody sandwich enzyme-linked immunosorbent assay (ELISA, BioVendor, Brno, Czech Republic) using two different monoclonal antibodies against human afamin as modified from a previously described protocol [29]. Recombinantly expressed and purified human afamin served as assay standard. According to the manufacturer’s manual, within-run and run-to-run coefficients of variation were 3.6 and 3.4%, respectively, at a mean afamin concentration of 80 mg/L.

Concentrations of PAPP-A and PlGF were measured with automated fluorescence assays (BRAHMS PAPP-A, 866.075, PlGF plus, 859.075) on a BRAHMS KRYPTOR immune analyzer (BRAHMS Kryptor Compact, Thermo Fischer Scientific, BRAHMS GmbH, Henningsdorf, Germany) in the Department for Gynaecology and Obstetrics at the University of Essen, Germany.

### Statistical analyses

Mann–Whitney *U* test or *t* test was performed to compare the parameters of interest between subgroups. Logistic regression was used to detect associations between afamin concentrations in the first trimester and subsequent PE. BMI and gestational age at blood sampling were included in the analysis as potential confounders, since afamin concentrations increase with gestational age [27] and BMI [17] as does the risk for PE. Afamin seems not to be synthesized by the placenta [27] and there is no evidence for a direct influence on afamin secretion through placental-derived substances. Thus, BMI and gestational age, but not PIGF and PAPP-A, were considered as confounders in the analysis.

Receiver operating characteristic (ROC) analysis was used to test the ability of serum afamin concentrations to discriminate between pregnant women developing PE and those not developing PE. All statistical analyses were performed with the R statistical package, version 3.4.0 [30].

## Results

### Serum afamin concentrations determined during the first trimester

Patient characteristics are shown in Table 1. Patients suffering from PE and women with uncomplicated pregnancies differed significantly regarding BMI, PIGF, gestational age at delivery and newborn weight.

**Fig. 1:**
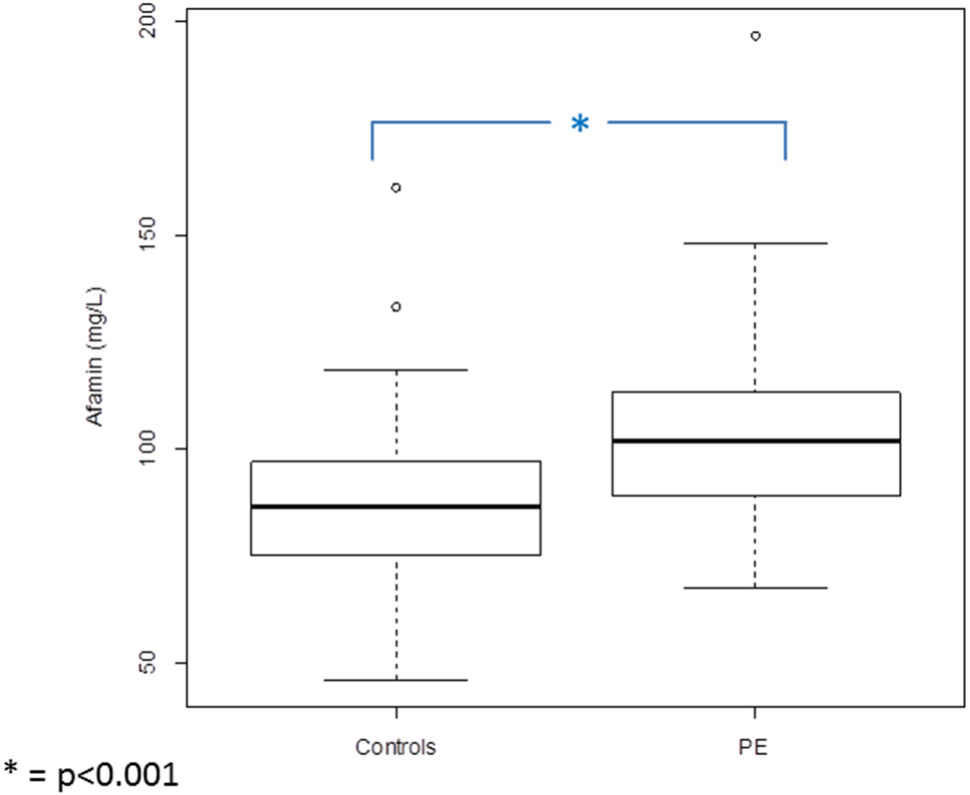
Boxplots illustrating the distribution of afamin concentrations in patients in the first trimester of pregnancy developing PE during pregnancy (*n* = 39) compared to control subjects without pregnancy complications (*n* = 98)

Median serum afamin concentrations during the first trimester in patients developing PE were significantly higher (median 101.81 mg/L; interquartile range (IQR) 88.94–113.26) than those in the control group (median 86.40; IQR 75.26–96.92; *p* < 0.001) (Fig. 1). After adjusting this difference for gestational age at blood sampling and BMI, afamin concentrations remained higher in patients developing PE than in the control group [odds ratio per afamin standard deviation: 1.60 (95% CI: 1.04–2.58; *p* = 0.04)].

**Fig. 2:**
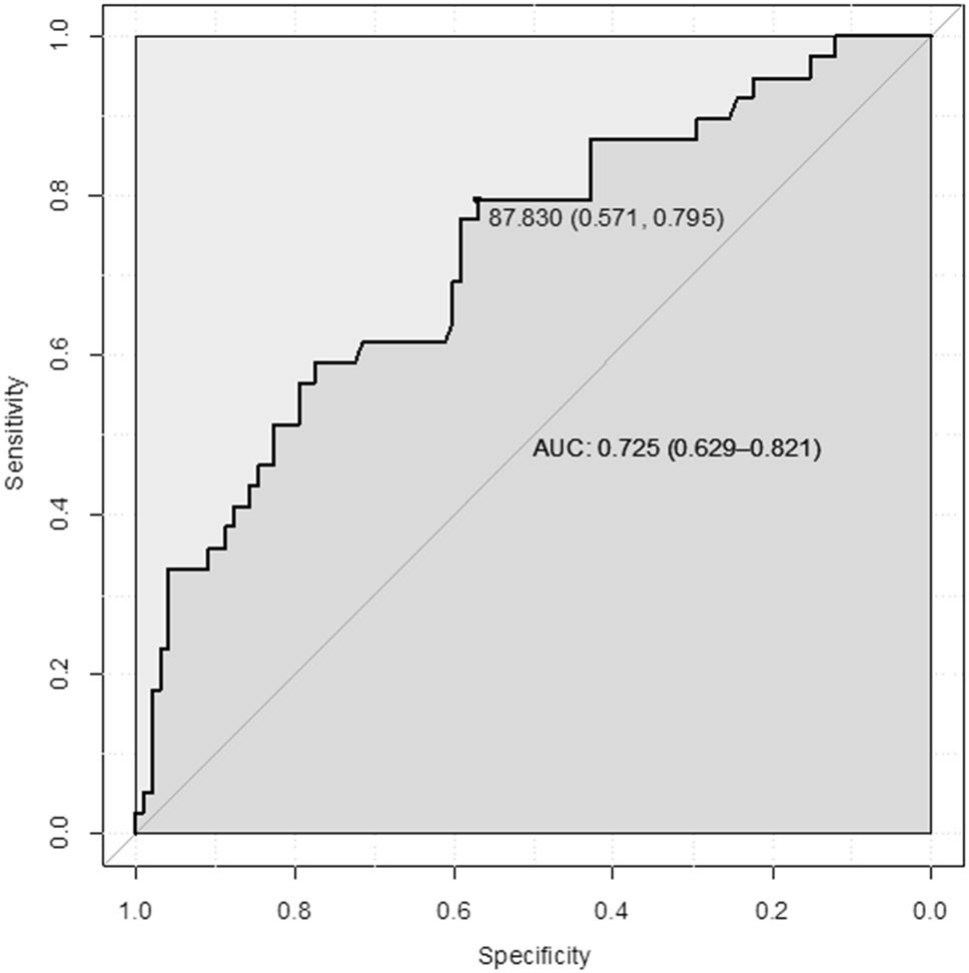
Receiver operating characteristics (ROC) analysis demonstrating specificity and sensitivity of afamin concentrations measured during the first trimester to discriminate between patients developing PE and women without pregnancy complications

Receiver operating characteristic (ROC) analysis showed that a serum afamin concentration of 87.8 mg/L had the best sensitivity (79.5%) and specificity (57.1%) to discriminate between women in whom PE will develop during their pregnancy and those with uncomplicated pregnancy. The area under the curve (AUC) was 0.73 (95% CI: 0.63–0.82) (Fig. 2).

### Subgroup analysis of patients who developed early-onset and late-onset PE

Patient characteristics of both subgroups are shown in Table 1. Subgroups differed significantly between gestational age at delivery and newborn weight and with borderline significance between age and PAPP-A.

**Fig. 3:**
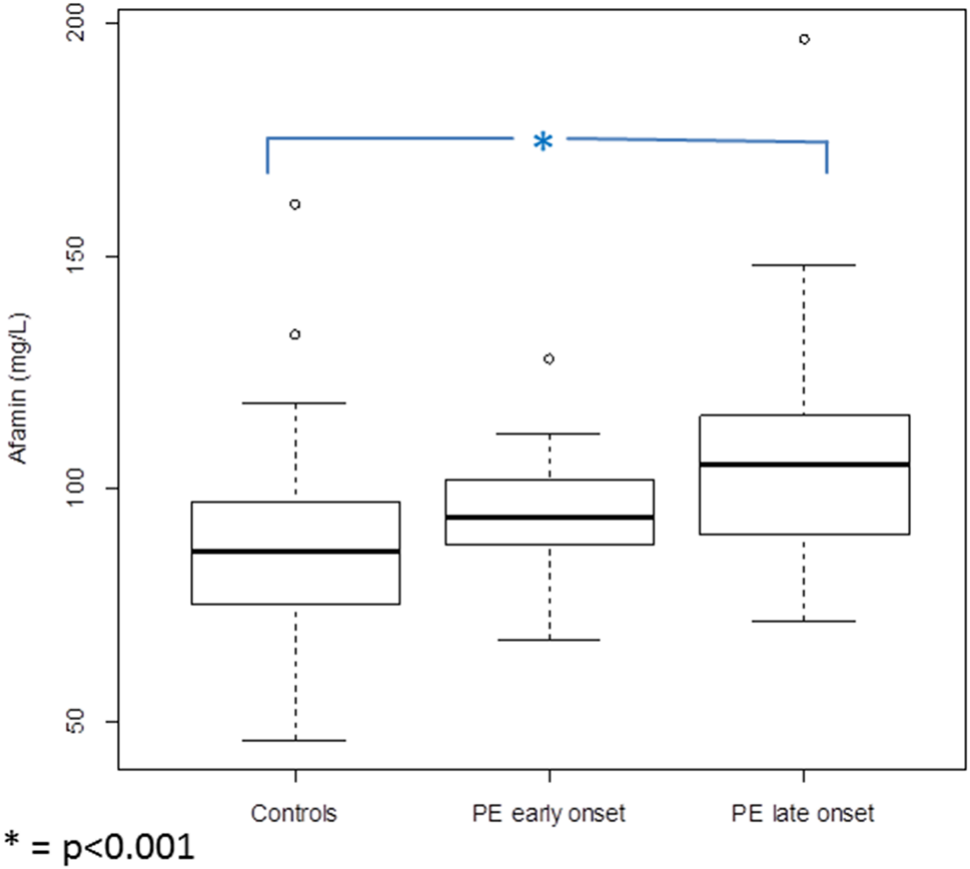
Boxplots illustrating the distribution of afamin concentrations in patients in the first trimester of pregnancy developing early-onset PE (*n* = 10) and late-onset PE (*n* = 29) during pregnancy compared to control subjects without pregnancy complications (*n* = 98)

Afamin concentrations were highest in women developing late-onset PE (median 105.10; IQR 89.78–116.17), followed by levels of women developing early-onset PE (median 93.68; IQR 88.03–102.00) and lowest in women without PE (median 86.40; IQR 75.26–96.92) (Fig. 3). Afamin values differed statistically differently between women who developed late-onset PE and controls (*p* < 0.001). The difference between afamin concentrations in women who developed late-onset and early-onset PE and between early-onset PE and controls was not statistically different (*p* = 0.20 and *p* = 0.10, respectively).

### Late-onset PE

After adjusting these differences for gestational age at blood sampling and BMI, afamin concentrations remained higher in patients developing late-onset PE than in the control group [odds ratio per afamin standard deviation: 1.62 (95% CI: 0.98–2.70; *p* = 0.06)]. Although statistical significance was not reached, the odds ratio remained identical compared to the analysis of the whole cohort (early- and late-onset PE).

**Fig. 4:**
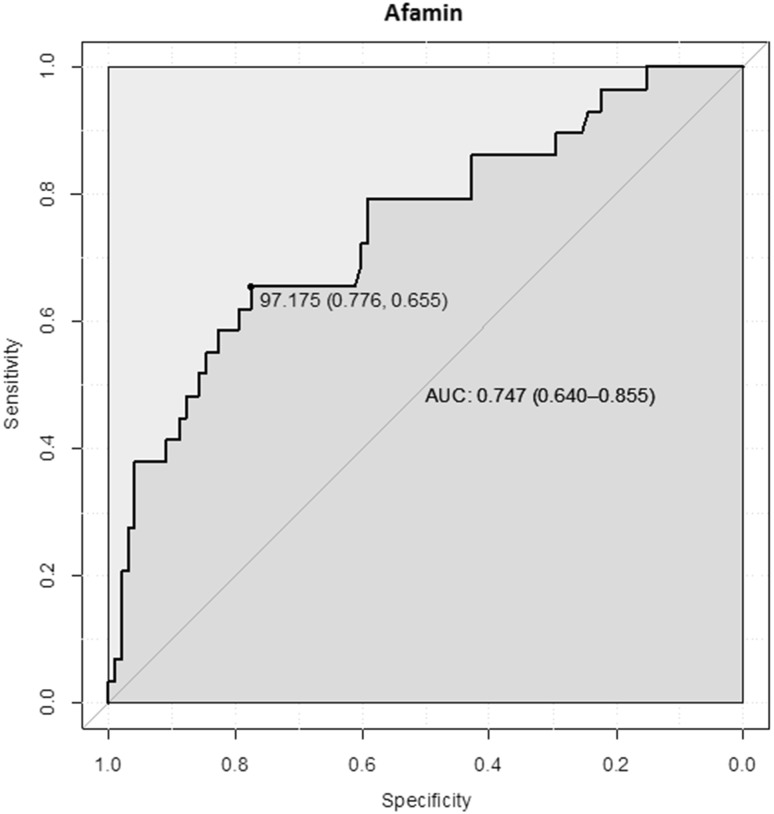
Receiver operating characteristics (ROC) analysis demonstrating specificity and sensitivity of afamin concentrations measured during the first trimester to discriminate between patients developing late-onset PE and women without pregnancy complications

ROC analysis showed that a serum afamin concentration of 97.2 mg/L had the best sensitivity (65.5%) and specificity (77.6%) to discriminate between women in whom late-onset PE will develop during pregnancy and those with uncomplicated pregnancy. The area under the curve (AUC) was 0.75 (95% CI: 0.64–0.86) (Fig. 4).

### Early-onset PE

After adjusting the results for gestational age and BMI, afamin concentrations remained only slightly higher in patients with developing early-onset PE than in the control group [odds ratio per afamin standard deviation 1.18 (95% CI: 0.61–2.27; *p* = 0.62)].

**Fig. 5:**
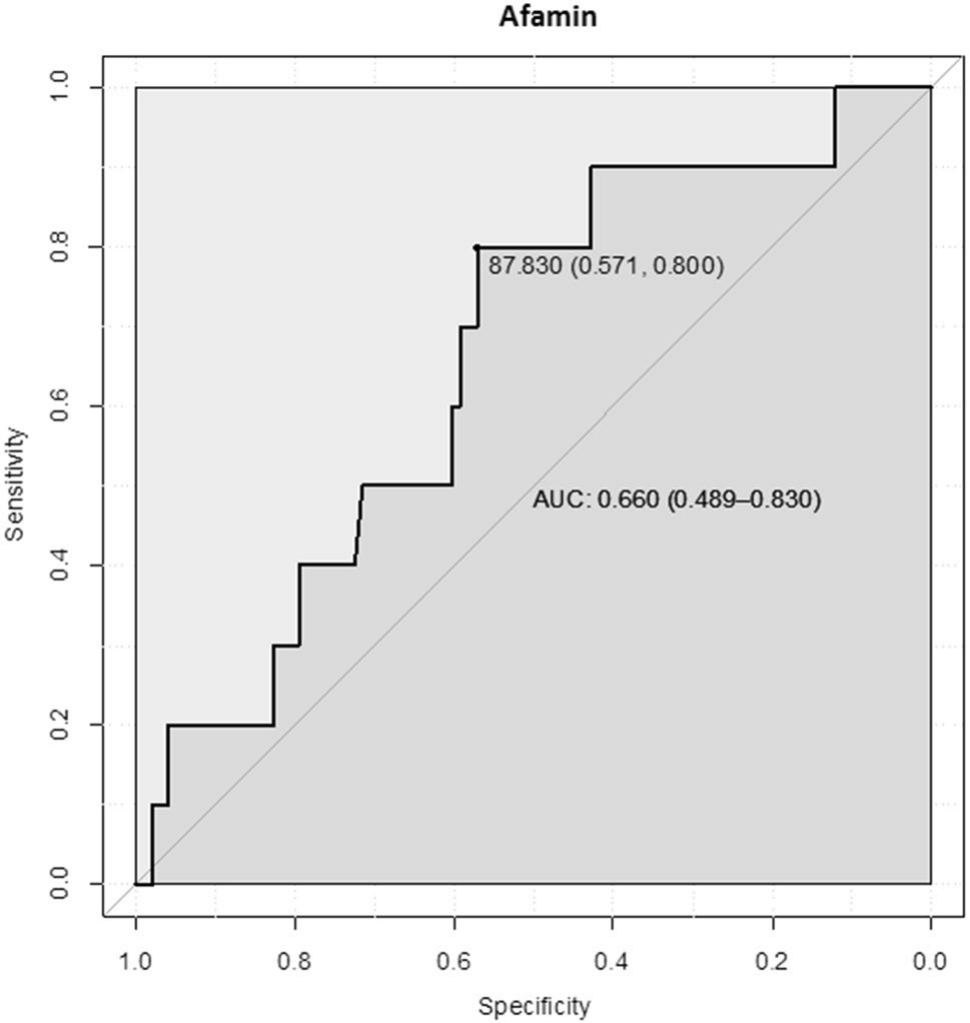
Receiver operating characteristics (ROC) analysis demonstrating specificity and sensitivity of afamin concentrations measured during the first trimester to discriminate between patients developing early-onset PE and women without pregnancy complications

ROC analysis showed that a serum afamin concentration of 87.8 mg/L had the best sensitivity (80.0%) and specificity (57.1%) to discriminate between women in whom early-onset PE will develop during their pregnancy and those with uncomplicated pregnancy. The area under the curve (AUC) was 0.66 (95% CI: 0.49–0.83) (Fig. 5).

Choosing a cut-off value of 106.9 mg/L and accepting a false-positive rate of 10%, the detection rate of all PE cases was 35.9%; of late-onset PE, it was 41.4% and of early-onset PE 20%, respectively.

## Discussion

In the present study, we compared serum afamin concentrations measured in the first trimester in 39 pregnant women developing PE in the course of their pregnancy with those of 98 women with uncomplicated pregnancies. Afamin concentrations were significantly higher in patients developing PE than in healthy control subjects. An afamin threshold concentration of 87.8 mg/L exhibited a sensitivity and specificity of 79.5 and 57.1%, respectively, discriminating between those two groups. In the study cohort, a high percentage of women (75%) developed late-onset PE. Subgroup analysis revealed that patients developing late-onset PE, but not those developing early-onset PE, showed substantially higher afamin concentrations compared to controls in the first trimester.

Our work confirms and extends a recently published study [22] also reporting significantly increased afamin concentrations in the first trimester in patients with developing PE compared to control subjects. The strength of our analysis is the subgroup analysis regarding late- and early-onset PE and the higher number of preeclamptic patients.

In accordance with the previous studies of our working group [19, 21], we found generally higher afamin concentrations in pregnant as well as in non-pregnant women compared to other study cohorts. This phenomenon remains somehow inexplicable and we hypothesise that ethnic differences might contribute to the higher concentrations in our study population.

Afamin is thought to reflect a condition of oxidative stress, since elevated concentrations correlate with features of the metabolic syndrome, especially IR and T2DM [17, 19]. Afamin concentrations were also reported to be positively correlated with IR in PCOS patients [19]. Oxidative stress, however, seems to be a concomitant factor in metabolic syndrome, IR and PCOS [31–33]. It was already demonstrated that preeclampsia is a status of elevated oxidative stress [10, 34]. Additionally, patients with placental dysfunction are at high risk to develop metabolic syndrome, IR [8] and cardiovascular disease later in their life [35].

75% of our preeclamptic patients, examined in the first trimester, developed late-onset PE. It was hypothesised that the pathogenesis of late-onset PE differs from the early-onset form. Early-onset PE seems to be caused by disturbed trophoblast invasion and the late-onset form rather results from adverse maternal conditions [7, 36]. Redman et al. hypothesised that late-onset PE derives from a limited intervillous perfusion resulting in intravillous hypoxia, oxidative stress and cell damage [6]. In conclusion, elevated afamin values may indicate a status of maternal oxidative stress which could mainly limit the placental function in the last trimester of pregnancy rather than disturbed early trophoblast invasion. Since afamin is not secreted by the placenta [27], elevated afamin concentrations more likely reflect a maternal contribution to the pathophysiology, e.g. a limited maternal vascular capacity [7].

The different pathophysiology is also reflected by the potency to predict and prevent placental disorders during the first trimester. Only early-onset PE can be predicted during the first trimester [4] and prevented by Aspirin^®^ intake at a > 90% percentage [2, 3]. In contrast, the number of PE cases of all trimesters which require delivery before the 42nd gestational week is predictable only in 54% of cases using biochemical and clinical parameters [37]. In our study, the detection rate of all PE cases (early- and late onset PE) was 36% using a cut-off value of 106.9 mg/L, with a false-positive rate of 10%. Regarding the late-onset PE cases only, the detection rate was 41.4%, respectively, using the same cut-off value and accepting a false-positive rate of 10%. This result is comparable to the predictive power of PIGF as a single parameter determined during the first trimester, which allows to detect 40% of PE cases by accepting a false-positive rate of 10% [37]. Further research with higher study numbers is recommended to build an algorithm which refers to a substantially higher detection rate of afamin concentrations by additional inclusion of the patient´s history and clinical features.

Because our early-onset PE subgroup was very small, our results cannot finally exclude an important role of oxidative stress in the pathogenesis of early-onset PE. Furthermore, clinical experience elucidates that the early- and the late-onset PE cannot be separated strictly and overlapping pathophysiological mechanisms are most likely. Physiological oxidative stress is an important component of normal foetal and placental development [38]. With placental maturation at the end of the first trimester, the oxygen level increases and reactive oxygen species (ROS) are produced. ROS themselves are responsible for an adequate trophoblast regression to cause the discoid placenta. Overproduction of ROS is associated with accelerated trophoblast regression resulting in placental dysfunction [39, 40]. In conclusion, the increased afamin concentrations found in first-trimester patients may reflect the present oxidative stress, which also could influence the placental differentiation at this step.

## Conclusion

As a consequence of our study, afamin concentrations determined in the first trimester may identify women at high risk of pregnancy complications associated with maternal oxidative stress and chronic inflammation such as PE. Elevated afamin values are hypothesised to reflect a maternal rather than a placental condition. Therefore, it is not surprising that elevated afamin concentrations identify women developing late-onset PE. Since afamin concentrations are independent of age, fasting state and daily fluctuations [29], the determination appears to indicate a very suitable and inexpensive new biomarker.

Since afamin concentrations mainly reflect a maternal condition, further research is recommended to explore the association between afamin determined before conception and the occurrence of pregnancy complications. Such a pre-conception risk profiling of women who want to get pregnant may offer an effective prevention of pregnancy complications by life-style modification or tailored early pregnancy care.
